# A Mixed Methods Study of Barriers to Help-Seeking for Intimate Partner Aggression in the LGBTQIA+ Community

**DOI:** 10.1177/08862605241270045

**Published:** 2024-08-26

**Authors:** Chelsea R. D’Cruz, Matthew D. Hammond, Louise Dixon

**Affiliations:** 1 Victoria University of Wellington, New Zealand

**Keywords:** IPA, intimate partner violence, gender, sexuality, support, prevention

## Abstract

People in the LGBTQIA+ community (i.e., lesbian, gay, bisexual, transgender, queer, intersex, asexual, and other gender/sexual minorities) experience greater rates of intimate partner aggression (IPA) than the general population and have fewer help-seeking pathways available. The current research examined the extent to which LGBTQIA+ people’s perceptions of barriers to help-seeking were associated with perceptions of societal heteronormativity—the belief that being cisgender and heterosexual is the norm—and whether the source of support was formal (e.g., police, counselors) versus informal (e.g., friends, family). The current research was conducted in two parts. In the first part of the study (Study 1a), structural equation modeling indicated a significant positive association between perceived societal heteronormativity and self-focused barriers (e.g., feeling too ashamed or guilty to seek help) but not with other-focused barriers (e.g., expecting unfair treatment). Instead, LGBTQIA+ people perceived greater other-focused barriers when considering formal compared to informal sources of support. In the second part of the study (Study 1b), we interviewed 10 LGBTQIA+ people about barriers to help-seeking for IPA. A reflexive thematic analysis identified four themes: (1) Who can hold the status of being a “victim”?; (2) The heightened importance of autonomy; (3) Formal supports need LGBTQIA+ competency; and (4) Judged by the outside in. The themes illustrated unique barriers experienced by LGBTQIA+ people when judging possible harm, choosing whether to seek help, and actual help-seeking. Altogether, current help-seeking pathways for IPA are generally inaccessible to people in the LGBTQIA+ community. IPA interventions for the LGBTQIA+ community require awareness of stigma, improved education for informal and formal support pathways, and the development of community-led interventions.

Intimate partner aggression (IPA) encompasses behaviors that are aggressive or controlling, directed toward a past or current intimate partner (Dixon & Wride, 2021), and predicts poorer psychological health, physical health, and behavioral outcomes (Laskey et al., 2019). Many interventions have been developed to help the estimated 27% of women worldwide who have experienced IPA and/or sexual violence in their lifetime (Sardinha et al., 2022). Rates of experienced IPA are even higher for people who are lesbian, gay, bisexual, transgender, queer, intersex, asexual, and other gender/sexuality minorities (LGBTQIA+) (see Salomaa & Matsick, 2019, for further definitions; Gover et al., 2015). For example, considering the current study in Aotearoa New Zealand, 17% of adults in general samples have experienced IPA, but this statistic is doubled for people in the LGBTQIA+ community (37%) (Ministry of Justice, 2022). Thus, LGBTQIA+ people are at a greater risk for IPA but potentially face distinct and additional barriers to seeking help. For example, heteronormative misconceptualizations of IPA as violence toward a female victim by a male perpetrator exclude people with diverse genders, sex characteristics, sexualities, and relationship structures (Baker et al., 2013). As such, recruiting a sample of LGBTQIA+ people is needed to address biases in research that has been based on cisgender heterosexual women (i.e., women who identify with their sex assigned at birth and are attracted to only men). In the current research, we surveyed 363 LGBTQIA+ people (Study 1a) and interviewed 10 LGBTQIA+ people (Study 1b) to understand their perceptions of societal heteronormativity and barriers to help-seeking for IPA.

## IPA Help-Seeking Among LGBTQIA+ People

Many people face barriers when seeking help for IPA, but those barriers are likely magnified for LGBTQIA+ people. General theorized barriers include social norms that limit people’s personal capacity to seek support (e.g., cultural values that treat help-seeking as shameful), limited funding for IPA resources, and stigmatization of victimization (Gover et al., 2015). However, theory on barriers often considers victims of IPA as they are commonly portrayed in media—cisgender heterosexual women who fit traditional gender norms of passiveness and femininity (Santoniccolo et al., 2021; Shelton, 2018). LGBTQIA+ people may not fit the common expectations of victims (Gover et al., 2015; Laskey et al., 2019), and thus common barriers are likely distinct for LGBTQIA+ people (Lelaurain et al., 2017). For instance, support programs are often based on paradigms with insufficient knowledge about LGBTQIA+ people (Guadalupe-Diaz & Jasinski, 2017; Messinger et al., 2021). LGBTQIA+ people interviewed about support services expressed fears about discrimination and exclusion by services for not presenting as a “typical” victim (Guadalupe-Diaz & Jasinski, 2017). Furthermore, LGBTQIA+ people can experience unique forms of IPA, such as a partner controlling their gender-affirming tools (e.g., hormones, binders, makeup) or “outing” them (i.e., disclosing their LGBTQIA+ status without consent) (Laskey et al., 2019), which are not commonly portrayed as IPA, so are less recognizable as “aggression” (Morgan et al., 2016). Finally, support services often require people to disclose their gender/sexuality and thus involve distinct risks of losing privacy and/or stigmatization (Guadalupe-Diaz & Jasinski, 2017; Morgan et al., 2016). In sum, LGBTQIA+ people are likely to experience greater, and perhaps qualitatively distinct, barriers when seeking help for IPA.

One possible factor underlying LGBTQIA+ people’s experienced barriers to help-seeking is the heteronormative lens taken by support services. *Heteronormativity* describes the social, cultural, and legal structures that enforce beliefs that there are only two genders, that gender is determined by biological sex, and/or that acceptable attraction occurs between men and women (Habarth, 2015). As such, heteronormativity is a form of stigma that many LGBTQIA+ people face daily. To illustrate, formal services often label themselves as being for women and offer help under the expectation that aggressors were their male partners (e.g., “women's shelters/refuges”) (Shelton, 2018), and thus do not explicitly label themselves for, or offer help to, LGBTQIA+ people. If LGBTQIA+ people view formal support agencies (e.g., refuges, doctors, counselors) as being inadequately knowledgeable or overtly discriminatory about LGBTQIA+ people (i.e., perceive society as holding heteronormative beliefs), then they will be unlikely to seek support for IPA. Thus, we hypothesized that LGBTQIA+ people who perceived society to be more heteronormative would perceive greater barriers to help-seeking for IPA.

Our research also considered that the barriers to help-seeking for LGBTQIA+ populations could depend on where people seek support. Theories on help-seeking, such as the help-seeking process framework (Liang et al., 2005) and the intimate partner violence stigmatization model (Overstreet & Quinn, 2013), specify that the help-seeking process begins with the choice of seeking formal or informal support. *Formal* sources encompass privatized support systems and services that have specialized training for IPA (e.g., counselors, medical professionals, domestic violence organizations, law enforcement) relative to *informal* sources that are known personally (e.g., family, friends, co-workers) (see Gover et al., 2015). According to the theoretical models of help-seeking, people’s choice between pursuing formal and/or informal support is informed by their expectations about how they will be treated by the chosen support source. In general, people tend to expect informal sources to be more supportive and helpful, and thus regardless of gender/sexuality, people commonly seek support from informal sources where those expectations are heightened compared to seeking support via formal sources (Sylaska & Edwards, 2014).

LGBTQIA+ people typically see formal support sources as unaccepting to their community (Guadalupe-Diaz & Jasinski, 2017), less available or accessible (Harden et al., 2022), and requiring potentially uncomfortable disclosure of their gender/sexuality (Edwards et al., 2015). For example, a study with transgender women found they felt less welcome in “women’s” refuges than cisgender women (Guadalupe-Diaz & Jasinski, 2017) and other research (Lelaurain et al.,2017) has indicated that LGBTQIA+ men are often excluded from “women’s refuge” services entirely. Furthermore, Donovan and Barnes (2020) interviewed LGBT people who had experienced IPA and described heteronormativity as important in their experiences of IPA (e.g., through beliefs about femininity being associated with victimhood). Collectively, these studies suggest that LGBTQIA+ people’s choice of seeking (vs avoiding) support for IPA is based on expectations of those sources holding heteronormative views that render any support inaccessible or as sources of additional harm. However, no research on IPA help-seeking has indexed LGBTQIA+ people’s perceptions about other people holding heteronormative views *or* examined the extent to which those perceptions predict their choice when comparing formal and informal sources of support. In the current research, we specifically examine the extent to which LGBTQIA+ people’s perceptions of greater levels of societal heteronormativity are connected to greater perceived barriers to help-seeking for IPA, and the extent to which these connections may differ when LGBTQIA+ people consider seeking help from formal (vs informal) sources.

## Current Research

LGBTQIA+ people experience significant barriers when seeking support for IPA, such as inaccessible support (Edwards et al., 2015), lower representation of IPA in LGBTQIA people’s relationships (Rollè et al., 2018), and the compounding stigma toward gender/sexuality with stigmatization of victims of IPA (Morgan et al., 2016). Many studies suggest that LGBTQIA+ people who perceive that society holds more heteronormative attitudes should experience greater barriers to help-seeking for IPA (e.g., experiencing negative treatment from support sources); however, this link has not yet been established. The current research was conducted in Aotearoa New Zealand. Aotearoa New Zealand is a bicultural country in Australasia. Aotearoa New Zealand is relatively egalitarian and accepting of LGBTQIA+ people relative to other countries (Equaldex, 2022), including the decriminalization of same-gender relations in 1986 and marriage equality laws established in 2013 (Carroll & Mendos, 2017). However, LGBTQIA+ people in Aotearoa New Zealand still experience inequalities compared to their cisgender heterosexual counterparts (e.g., greater mental health difficulties, lower disposable income) (Stats NZ, 2021) and particularly when considering intersectionality for those harmed by enduring colonial practices and ideologies on Māori—the Indigenous peoples of Aotearoa New Zealand (see Le Grice et al., 2022). Hence, our results inform help-seeking for IPA within a context in which people with minoritized sexualities and genders have relatively higher legal protections and societal acceptance, and yet still encounter exclusion from support services.

We conducted a mixed-methods investigation of LGBTQIA+ people’s perceptions of societal heteronormativity and their expected barriers to help-seeking for IPA. In Study 1a, we surveyed 363 LGBTQIA+ people. Structural equation models tested the extent to which people’s perceived societal heteronormativity simultaneously predicted greater (1) self-focused barriers to help-seeking (e.g., feelings of shame and embarrassment) and (2) other-focused barriers to help-seeking (e.g., expected discrimination when seeking support), moderated by the context of formal (e.g., doctors or police) versus informal (e.g., friends or family) sources of support. Following the help-seeking process framework (Liang et al., 2005) and the intimate partner violence stigmatization model (Overstreet & Quinn, 2013), we hypothesized that LGBTQIA+ people’s perceptions of greater societal heteronormativity would be associated with experiencing greater barriers to help-seeking for IPA (Hypothesis 1). Furthermore, building on research that suggests formal sources of support are particularly inaccessible to LGBTQIA+ people (Harden et al., 2022), we hypothesized that the positive association between societal heteronormativity and barriers to help-seeking would be magnified for formal (vs informal) sources of support (Hypothesis 2). In Study 1b, we considered that the experiences of IPA and barriers to help-seeking can be unique to LGBTQIA+ people (Barrett & Sheridan, 2017; Shelton, 2018), and so conducted interviews with 10 LGBTQIA+ people for a reflexive thematic analysis (Braun & Clarke, 2022). The themes answered the following research question: “What factors do LGBTQIA+ people in Aotearoa New Zealand perceive to be barriers and facilitators to help-seeking for IPA?”

## Study 1a

### Method

#### Procedure and Participants

A pre hoc power analysis in G*Power (Faul et al., 2009) identified that 241 participants provided power of .90 to detect a medium-sized effect in our planned model. Participants opted into a survey hosted on Qualtrics from advertisements posted around university campuses, social media (e.g., the local Transgender and Intersex Facebook group), and local LGBTQIA+ organizations (e.g., InsideOUT) and entered a draw for one of five $50 supermarket vouchers. We stopped data collection the month after we reached our target sample size to account for any potential exclusions. Data were collected from 367 adults in Aotearoa New Zealand. Four participants were excluded due to opting for their “response to be deleted” at the end of the survey. The study received ethical approval from the Victoria University of Wellington Human Ethics Committee (#30025). The analysis was pre-registered (https://osf.io/bk93y/).

Demographic information for the final sample (*N* = 363) is shown in Table 1. Participants identified diverse genders and sexualities; the largest groups were women (*N* = 140; 38.6%) and people who were bisexual/pansexual (*N* = 122; 33.6%). In addition, 78 (21.5%) participants were transgender, and 22 (6.1%) participants were unsure if they were transgender. Five (1.4%) participants indicated they had a variation in sex characteristics (i.e., intersex), and 30 (8.3%) participants were unsure if this applied to them. Most participants were in the age groups of 18 to 24 (*N* = 140; 38.6%) or 25 to 34 (*N* = 85; 23.4%).

**Table 1.** Demographic Information for Study 1a.

**Gender**							
Woman	Nonbinary		Man		Self-Described		Takatāpui^a^
140 (38.6%)	72 (19.8%)		53 (14.6%)		33 (9.1%)		11 (3.0%)
**Sexuality**							
Bisexual/pansexual	Lesbian	Gay	Queer	Asexual	Heterosexual	Takatāpui^a^	Self-Described
122 (33.6%)	76 (20.9%)	37 (10.2%)	27 (7.4%)	21 (5.8%)	18 (5.0%)	15 (4.1%)	14 (3.9%)
**Ethnicity**							
NZ European/Pākehā	Māori		Other		Pasifika		Asian
213 (58.7%)	29 (8.0%)		18 (5.0%)		12 (3.3%)		11 (3.0%)

*Note*. Frequencies and percentages will not total the sample size because some participants selected multiple options, and not all participants filled out the demographic information.

a “Takatāpui” refers to Māori (Indigenous peoples of Aotearoa New Zealand) who identify with minority genders and sexualities (Le Grice et al., 2022).

### Materials

#### Perceived Societal Heteronormativity

Participants completed a version of the Heteronormativity Attitudes and Beliefs Scale (Habarth, 2015) adapted to assess perceptions of societal heteronormativity. Following Fiske et al.’s (2002) measurement of perceived societal stereotypes, participants rated how they thought *most people in society* would answer 16 items indexing heteronormative beliefs (e.g., “All people are either male or female,” “Gender is the same thing as sex,” and “Sex is complex; in fact, there might even be more than 2 sexes [reverse-coded]”; 1 = *strongly disagree*, 7 = *strongly agree*). Items were averaged so that higher scores indexed participants’ higher perceptions that people in society held heteronormative beliefs (α = .95).

#### Barriers to Help-Seeking for IPA

We selected items for three theorized barriers to help-seeking for IPA from the Barriers to Help Seeking Scale (four items) (Mansfield et al., 2005) and the Barriers to Mental Health Care Measure (11 items; Umubyeyi et al., 2016; 1 = *strongly disagree*, 7 = *strongly agree*). Participants completed the items twice (counterbalanced across participants), once directed to consider seeking help from *formal* sources of support (“If you were to seek help from a formal source of support for a situation like this, please rate how much of a reason each item would be to not seek help for the situation described above”) and the other directed to consider seeking help from *informal* sources of support.

Our pre-registered hypothesis was that the items would be grouped into three scales indexing stigma (α = .73), privacy concerns (α = .66), and knowledge about help sources (α = .60). However, due to the low reliabilities for these scales, we conducted a post hoc principal components analysis (PCA) in jamovi version 2.3 (The Jamovi Project, 2022). The PCA indicated that participants’ responses were grouped into two components (see Supplemental Table S1 for the full results). One component—*other-focused barriers—*consisted of nine items of negative treatment from support providers (e.g., “I would not believe that I would get proper help”; “I believe I would not be treated fairly because of my sexuality”). The other eight items were *self-focused barriers*—participants’ intrapersonal negativity about help-seeking (e.g., “It would seem weak to ask for help”; “I would be ashamed to show others how troubled I was”). Items were averaged so that higher scores indexed greater perceptions of *self-focused* (α = .773) or *other-focused* (α = .791) barriers.

### Results

We tested our hypotheses in a model that included the two outcomes simultaneously to account for the tendency for people who perceived greater self-focused barriers to help-seeking to also perceive greater other-focused barriers (*r* = .38, *p* *<* .01 for formal supports; *r* = .45, *p* *<* .01 for informal supports; see Supplemental Table S2 for full descriptive statistics and correlations). We used multilevel structural equation modeling using Mplus version 8.4 (Muthén & Muthén, 1998-2017), specifying a random-intercepts model with full-information maximum likelihood estimation, to account for the measurement dependency of participants’ ratings of the same barriers for formal versus informal contexts. We simultaneously regressed the two barriers to help-seeking (i.e., self-focused and other-focused) on perceived heteronormativity, moderated by whether help-seeking was from a formal or an informal source (coded as 0 = *informal*, 1 = *formal*). Continuous predictors were grand-mean centered. The model was fully saturated so we do not report fit statistics.

**Table 2.** Study 1b Constructed Themes.

Theme	Description
Summary	
1. Who can hold the status of being a “victim”? a. Hegemonic gender roles limit LGBTQIA+ experiences of IPA b. People feel responsible for maintaining positive appearances c. Education is needed to facilitate LGBTQIA+ people’s identification with IPA	Preconceived notions of IPA and the LGBTQIA+ community limit identification and acknowledgment of IPA in LGBTQIA+ relationships, which restricts help-seeking.
2. The heightened importance of autonomy a. The unknown within the help-seeking process is risky for LGBTQIA+ people b. Choice allows LGBTQIA+ people to seek help	Agency, autonomy, and privacy are needed when people seek help for IPA due to the extra risks LGBTQIA+ people face.
3. Formal supports need LGBTQIA+ competency a. Lack of understanding from formal services leads to lack of community trust b. LGBTQIA+ people perceive formal services as harmful	LGBTQIA+ people expect formal services to treat them differently and provide ineffective support.
4. Judged by the outside in a. Outness complicates the help-seeking process b. Effective help-seeking requires the LGBTQIA+ community to be accepted by society	Discrimination against the LGBTQIA+ community is isolating for LGBTQIA+ people who experience IPA, and efforts to promote LGBTQIA+ help-seeking will never be enough without true and full LGBTQIA+ acceptance.

*Note.* IPA = intimate partner aggression.

**Figure 1.**
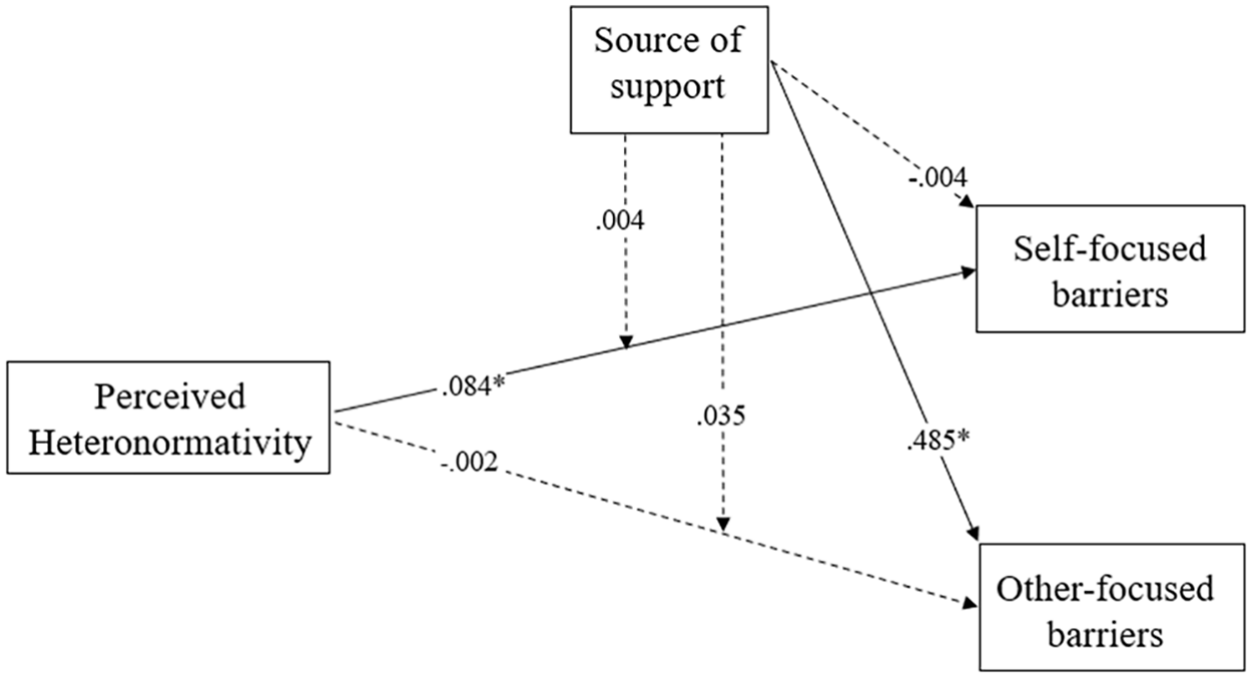
Structural equation model of the links between perceived heteronormativity and two types of barriers to help-seeking, moderated by whether those barriers occurred for formal (e.g., police) or informal (e.g., family) sources of support. *Note.* Dashed lines indicate non-significant relationships. *p < .05.

The results are depicted in Figure 1. Evidence indicated a small association between greater perceived heteronormativity and greater self-focused barriers to help-seeking (*B* = .084, 95% CI [0.004, 0.164], *t* = 2.063, *p* = .039), but no evidence emerged to suggest that perceived heteronormativity was associated with other-focused barriers to help-seeking (*B* = −.002, [−0.074, 0.069], *t* = −0.063, *p* = .950). On average, participants perceived greater other-focused barriers for formal (vs informal) sources of support (*B* = .485, [0.366, 0.604], *t* = 8.006, *p* < .005), but no differences emerged in perceived self-focused barriers (*B* = −.004, [−0.102, 0.094], *t* = −0.077, *p* = .939). Finally, no evidence emerged to suggest that these associations differed when participants were considering informal versus formal sources of support. Specifically, no significant interactions of source emerged for self-focused barriers (*B* = .004, [−0.063, 0.071], *t* = 0.106, *p* = .915) or other-focused barriers (*B* = .035, [−0.043, 0.112], *t* = 0.874, *p* = .328). In sum, supporting Hypothesis 1, participants’ greater perceived heteronormativity was related to perceiving more intrapersonal (self-focused) barriers. Although participants generally perceived formal support sources to have more other-focused barriers, no evidence emerged that the link between perceived heteronormativity and perceived barriers was heightened for formal sources (i.e., no evidence for Hypothesis 2).

## Study 1b

Study 1a identified a small association between perceived heteronormativity and self-focused barriers to help-seeking for IPA (e.g., avoiding help due to feeling too ashamed) and no evidence for other-focused barriers (e.g., avoiding help due to expecting discrimination). The direction of the significant result was consistent with the suggestion of prior research identifying perceived heteronormativity is associated with concerns about how their help-seeking would be perceived by others (Harden et al., 2022), but the partial evidence and relatively small effect size were inconsistent with the claim that perceived heteronormativity is fundamental to LGBTQIA+ help-seeking (Donovan & Barnes, 2020). Thus, the mixed findings suggested the need to consider the distinct experiences and expectations of LGBTQIA+ people more deeply in the context of seeking help for IPA. A qualitative approach gives voice to LGBTQIA+ people who have been historically underrepresented in research (Laskey et al., 2019) and thus provides insight into potential facets of heteronormativity and barriers to help-seeking for IPA that are not conceptualized by existing models of help-seeking. In Study 1b, we built on the survey findings by interviewing LGBTQIA+ people in Aotearoa New Zealand about the barriers and facilitators to help-seeking for IPA, including potential distinctions between formal and informal supports.

### Method

#### Participants and Procedure

Participants in Study 1a indicated if they would like to be interviewed for further discussion. We prioritized interviewing people with the highest scores of perceived heteronormativity, or who were Takatāpui (Indigenous peoples of Aotearoa New Zealand who identify with minority genders, sexualities, and sexes) (Le Grice et al., 2022), Māori, and/or transgender due to their underrepresentation in research. Thirty-two participants were invited to participate and 10 of those participants agreed to be interviewed. Two participants were gay men, three were lesbian or queer women, two were nonbinary, and one rejected gendered labels. Participants’ ages ranged from 17 to 39 (*M* = 27.20, *SD* = 6.49). In addition, two participants were transgender, and one was unsure if they were transgender.

The first author conducted semi-structured interviews online over Zoom from June to September 2022. Interviews ranged from 12 to 53 min (*M* = 26 min). The thematic analysis was conducted on question prompts that were relevant to perceptions of support sources for IPA for the *Rainbow community*—the label for LGBTQIA+ people in Aotearoa New Zealand? (see Supplemental Table S3 for all interview questions). Analyzed answers included factors that “prevent Rainbow people seeking help from formal services” or “help Rainbow people access services that could help with these experiences.” Interviews were transcribed verbatim by three transcribers and the first author reliability-checked each transcript. Participants each received a $20 supermarket voucher, were offered an interview summary, and were each given a pseudonym in the reporting of the results. Ethical approval was obtained from the Victoria University of Wellington Human Ethics Committee (#30025).

#### Positionality, Reflexivity, and Author Contributions

The first author (they/she) conducted the thematic analysis in consultation with the second and third authors, and is a young, queer, person of color from Aotearoa New Zealand. Their belonging to Aotearoa New Zealand’s LGBTQIA+ community affords insider knowledge about the nuanced way that gender and sexuality are experienced, including how these are often intertwined and inseparable. Specifically, they approached the current research with a focus on acknowledging intersectionality—that gender, sexuality, and ethnicity interact in unique ways for each person and thus produce experiences specific to how those groups overlap. The second author (he/him; a cisgender heterosexual NZ European/Pākehā man) and the third author (she/her; a cisgender heterosexual British woman) are academics with topic and methods expertise who provided reflection and feedback on the themes. In answering the research question, “What factors do LGBTQIA+ people in Aotearoa New Zealand perceive to be barriers and facilitators to help-seeking for IPA?,” four themes were constructed, summarized in Table 2, and detailed in the following sections.

### Reflexive Thematic Analysis

#### Theme 1. Who Can Hold the Status of Being a “Victim”?

Participants expressed that they perceived most people outside of the LGBTQIA+ community as holding heteronormative beliefs about relationships, gender, and IPA, which would exclude them from being considered “victims.” Specifically, participants discussed expectations and misconceptions about IPA, where women are seen only as victims and men are seen only as perpetrators. Participants explained that this gendered narrative of victimhood further complicates help-seeking when victims are expected to be women who adhere to hegemonic gender norms of femininity, and that this creates a conceptualization of an *ideal victim* that limits the capacity of LGBTQIA+ people to seek help. For example, participants expressed that they expected other people not to classify aggression between people of the same gender as IPA. Consequently, participants explained that they believed LGBTQIA+ people may be seen as unable to even *hold* a “victim” status. This is especially prevalent for men, with previous research highlighting the contradiction seen between masculinity and victimization (Dixon et al., 2023). Hegemonic expectations also limit the extent to which participants believed LGBTQIA+ people could communicate with others about their experiences of IPA. Quinn (Queer, non-gendered, *any/all* pronouns), an interview participant, explained that they did not want seeking help for their IPA experiences to result in invalidation of their partner’s gender: “I had concerns about people maybe having to make me deal with transmisogyny toward my ex-partner; I didn’t want the source of her behavior to be attributed to some kind of essential male.” These societal expectations about what it means to be a “man” or “woman” are opposing and mutually exclusive, with aggression framed as something only belonging to men. This limits the behaviors that can be identified as “aggression” within non-heteronormative relationships and contributes to transphobic notions surrounding transgender women and aggression.

LGBTQIA+ people in the interviews expressed being disconnected from typical ideas of victimhood as part of feeling responsible for maintaining a positive image in the community. Participants identified that LGBTQIA+ people might avoid actions that would portray their community in a negative light and, in turn, mean that LGBTQIA+ people who experience IPA feel disconnected from sources of support. Frankie (Queer woman, *she/her*) explained this: “There’s always that sense that you’ve gotta represent your minority.” Participants also expressed how the need to maintain appearances extended to not reporting aggression from their partners. Some participants who experienced IPA explained that they avoided seeking help to prevent their partner from being labeled as a perpetrator. Casey (Gay man, *he/him*) linked this fear of the demonization of perpetrators to the type of support people might choose: “I would think that most people would seek informal support services in the first instance, perhaps because it is their partner, they’re not necessarily wanting them to get into trouble.” Thus, while the fear of getting a partner in trouble may exist for all people who experience IPA, participants felt that LGBTQIA+ people may feel responsible for representing their community in addition to protecting their partner. This is further reinforced by analysis from Indigenous scholars (Le Grice et al., 2022) that outlines how dominant Eurocentric approaches prefer punishment of perpetrators over rehabilitation, which can increase barriers for Indigenous people in the LGBTQIA+ community. For example, some people who experience IPA may perceive that seeking support would necessitate reporting their partner, which could minimize opportunities to sustain their relationship in a healthier manner.

Finally, participants expressed that their disconnect from portrayals of being a “victim” stemmed from a lack of healthy but non-heteronormative relationship role models, which resulted in a limited understanding of appropriate relationship behaviors. Participants noted the challenge of identifying when a relationship is *not* healthy and the lack of available information on what IPA looks like when it does not adhere to a gendered narrative (see Santoniccolo et al., 2021). Frankie (Queer woman, *she/her*) explained that if gendered expectations are not challenged, they limit recognition of IPA in non-heteronormative relationships: “[there are] silences that exist when we don’t view women as being able to be violent to each other, and how if we can’t see it and it’s not verbalized to us that it’s possible, then we can’t even identify it in our own relationships.” Consequently, there is a need to educate people both in and outside of the LGBTQIA+ community about what aggression can look like for LGBTQIA+ people.

#### Theme 2.The Heightened Importance of Autonomy

When asked about the factors that would influence their choice to seek help for IPA, participants expressed the challenge of lacking information about the help-seeking process. Specifically, LGBTQIA+ people did not know what to expect from formal services when seeking help for IPA, including potential discrimination from those services. Casey (Gay man, *he/him*) described how even when a formal service may not discriminate against LGBTQIA+ people, there might still be fear that individuals within those services could still be discriminatory: “You would hope that if it was a formal service, that they would be, but formal services are staffed by people who have their own thing.” Here, Casey described how services may position themselves as being understanding and accepting of the LGBTQIA+ community, but individuals within those services may exhibit their own biases. As a result, LGBTQIA+ people must choose between the potential harm and benefits that result from seeking help. For some, this choice may depend on their perceptions of the severity of aggression. To illustrate, participants expressed willingness to risk the potential costs of seeking help if the aggression is perceived as “severe enough.” Sam (Queer, nonbinary, *they/them*) described how this can be a difficult choice for LGBTQIA+ people: “I think it’s all about risk assessment for queer people.”

Giving people choices about their help-seeking can reinstate the autonomy and agency that is lost through victimization, thus empowering people during the help-seeking process. This is illustrated in Bailey’s (Lesbian woman, *she/they*) explanation that help-seekers need autonomy when seeking help: “Quite often you need to be helped but you don’t know how, and you don’t know what options are out there. So, really giving people more control over their situation that they’re already feeling no control over.” The idea about the harm that results from a lack of choice over the help-seeking pathway (i.e., informal vs formal support) was prominent in participants’ discussions of how help-seeking differs for LGBTQIA+ people. Specifically, if LGBTQIA+ people have unaccepting informal support or no informal support, then they have no choice over which help-seeking route to take.

#### Theme 3.Formal Supports Need LGBTQIA+ Competency

Participants believed that formal support lacked necessary LGBTQIA+ competency and did not understand LGBTQIA+ relationships. Participants explained their experiences with general formal services (e.g., medical professionals) where they felt misunderstood due to heteronormative expectations, and this contributed to the belief that IPA-specific formal services would provide ineffective support for IPA within the LGBTQIA+ community. Participants believed that formal sources of support would see them as inferior to people in heteronormative relationships, and this would lead to them receiving ineffective support, as Morgan (Pansexual, nonbinary, *they/them*) explained: “If you are a queer person, particularly if you are transgender, there’s a fear around how your problem will be taken seriously compared to cis[gender] het[erosexual] people.” Consequently, participants lacked trust in formal services and believed that formal services are unable to provide LGBTQIA+ people with effective support for IPA when needed.

Participants perceived formal supports as operating from heteronormative conceptions of IPA, and thus as unsafe environments. Casey (Gay man, *he/him*) explained how the positioning and language of formal supports diminish safety for LGBTQIA+ people: “Thinking about trans and nonbinary people having to go to a place called ‘Women’s Refuge’ is problematic in and of itself.” The exclusion of the LGBTQIA+ community (especially men and transgender or nonbinary people) from formal sources informed participants’ expectations of the types of responses they were likely to get if they were to seek help for IPA from formal sources. Specifically, there was an overwhelming expectation that formal sources would discriminate against LGBTQIA+ people. Consequently, environments that are supposed to provide support are instead seen as contributing to harm.

Participants expressed mistrust of the police and saw the police as a source of harm. Frankie (Queer woman, *she/her*) explained how the harm caused by police and the distrust of people in the LGBTQIA+ community toward the police differs for people with intersecting identities. Here, Frankie highlighted the unequal treatment toward people who are Māori, and how this would stop her from seeking help from the police if she needed to:

The other thing that would almost entirely stop me from seeking formal help, is that my girlfriend is Māori, and myself as a Pākehā (white) person, is a huge power dynamic there, and going to the cops and then putting my Māori girlfriend into the ‘justice’ system just feels really icky to me.

Overall, participants held the belief that formal services would not keep LGBTQIA+ people safe, and in some instances, would inflict harm toward LGBTQIA+ people already experiencing harm within an intimate relationship.

#### Theme 4.Judged By the Outside In

Participants highlighted that if an LGBTQIA+ person was not “out,” then seeking support would require this disclosure. This is an issue for LGBTQIA+ people if those in their informal support circles are not accepting of the LGBTQIA+ community, or if formal support sources are not safe for LGBTQIA+ people. For example, Frankie (Queer woman, *she/her*) expressed that she would not want to seek formal support because of how exhausting the process of continually coming out would be, in addition to the added layer of worry about not being accepted: “I would expect to have to come out to the police; a constant, relentless, process of coming out.” By contrast, participants highlighted that if an LGBTQIA+ person was out to their support circles, then informal support sources would generally be more accepting and available than formal support sources. Morgan (Pansexual, nonbinary, *they/them*) explained the preference for informal sources: “[With] formal services, you don’t know if they’re going to treat you differently for who you are, whereas friends and family, you might expect a certain way they’ll treat you.” The process of continually coming out, or expecting to have to come out, compounds the pre-existing stressors in help-seeking, which leaves LGBTQIA+ people with a constant reminder that they do not fit heteronormative expectations.

Overall, participants indicated that acceptance of the LGBTQIA+ community within help-seeking contexts makes help-seeking easier. First, participants expressed that they would find it easier to talk to fellow LGBTQIA+ people if they were having issues within their relationships, due to sharing a lived experience. Ashley (Lesbian woman, *she/her*) explained this, saying, “We always feel judged by the outside in. So, I think that in these spaces, you need user-friendly people who are operating from a worldview which is usually based on lived experience.” Participants indicated that having LGBTQIA+ people within formal support services would allow formal services to provide support from an LGBTQIA+ perspective. Participants highlighted the emptiness and performativity of formal services’ claim of being “LGBTQIA+ friendly” when their language, processes, and foundations lack genuine understanding, care, and knowledge. As such, true inclusivity that comes from a service informed by lived experience is needed for formal sources of support to be effective for LGBTQIA+ people.

## Discussion

The current research investigated LGBTQIA+ people’s perceptions of barriers to seeking support for IPA in two studies: a survey (Study 1a) and interviews (Study 1b). We hypothesized that perceived societal heteronormativity would be positively associated with barriers to help-seeking for IPA. Study 1a, a survey of 363 LGBTQIA+ adults in Aotearoa New Zealand, indicated that people who perceived society to hold greater heteronormative beliefs also perceived heightened barriers to help-seeking that were self-focused (e.g., perceiving that seeking help would seem weak and would result in feelings of shame). Thus, evidence partially supported Hypothesis 1 in Study 1a: Perceived societal heteronormativity was associated with self-focused barriers but not other-focused barriers. Comparatively, Hypothesis 2 was not supported: There was no evidence in Study 1a that perceiving society to hold more heteronormative beliefs was linked with a heightened perception of barriers when seeking help from formal (vs informal) sources of support. Interviews with 10 LGBTQIA+ people in Study 1b indicated that participants viewed heteronormative narratives of IPA as a barrier to seeking help, in addition to uncertainty throughout the help-seeking process, a lack of accessible help sources, and general prejudice toward the LGBTQIA+ community. The current research suggested that LGBTQIA+ people experience distinct barriers that need to be considered in their help-seeking for IPA.

The survey (Study 1a) and interviews (Study 1b; Theme 3: Formal Supports Need LGBTQIA+ Competency) both identified that LGBTQIA+ people perceived many barriers to formal sources of support that were specific to qualities of those formal services (i.e., were other-focused barriers). These findings extend both the help-seeking process framework (Liang et al., 2005) and the intimate partner violence stigmatization model (Overstreet & Quinn, 2013). Both theories posit help-seeking as a stigmatizing process, where help-seekers must face stigma-related barriers throughout the help-seeking process. According to the intimate partner violence stigmatization model (Overstreet & Quinn, 2013), cultural stigma (i.e., social ideologies) is related to anticipated stigma (i.e., the stigma one expects to encounter) and internalized stigma (i.e., their stigmatizing self-views). The current research found evidence to suggest that perceiving higher levels of cultural stigma (i.e., societal heteronormativity) was associated with greater feelings of internalized stigma (i.e., self-focused barriers). However, Study 1a did not identify a relationship between cultural stigma (i.e., societal heteronormativity) and anticipated stigma (i.e., other-focused barriers). Comparatively, Study 1b identified various anticipated barriers to help-seeking that participants believed to stem from societal heteronormativity. Accordingly, the current research showed statistical support for the relationship between cultural stigma and internalized stigma, and qualitative (but not quantitative) support for the relationship between cultural stigma and anticipated stigma. In sum, both Study 1a and 1b indicated that (a) LGBTQIA+ people experience heightened barriers when seeking help for IPA and (b) their experiences of heteronormativity in society, such as misunderstanding of LGBTQIA+ relationships, would be a particular hindrance to receiving effective support.

### Implications for Theory on Help-Seeking

The results from both Study 1a and 1b supported the notion that stigma (e.g., societal heteronormativity) is integral to the help-seeking process for people in the LGBTQIA+ community who experience IPA. Stigma plays a substantial role in help-seeking (Overstreet & Quinn, 2013), and drawing on models of help-seeking (Liang et al., 2005), is likely to suppress help-seeking for LGBTQIA+ people at each stage of the help-seeking process (e.g., identifying a problem; deciding to seek help; and choosing support). Following Overstreet and Quinn (2013), our results indicate two distinct aspects of cultural stigma—ideological stigma and structural stigma—that are meaningful when LGBTQIA+ people seek help for IPA. On one side, ideological stigma was present in participants’ attitudes as help-seekers, limiting help-seeking by perpetuating discrimination, or endorsing feelings of weakness, shame, and embarrassment. On the other, structural stigma was present in the repeated message about the inaccessibility of support services. Specifically, even when formal sources of support may not express *ideological* stigma, participants noted the lack of education and resources that perpetuated structural stigma. Thus, even when people do not perceive high levels of societal heteronormativity, they may still expect to receive ineffective support due to structural inequalities in the inaccessibility of formal help services.

The current research also reiterates the harm of gendered narratives in which IPA is experienced by cisgender women in heterosexual relationships with male partners (see Laskey et al., 2019). To illustrate, in Theme 1, participants emphasized the compounded harm of their experienced aggression feeling silenced because it did not fit the gendered narrative, and the difficulties in seeking help for IPA when support services could see it in a gendered frame and perpetuate harm (e.g., a support service could misgender a partner by associating “aggressor” with being a man). Comparatively, gender-inclusive research on IPA shows that acknowledgment and understanding of the diversity of victims is beneficial to the recovery of men who experience IPA victimization (Dixon et al., 2023). Our results underscore the need to recognize when misconceptualizations of IPA can cause, or perpetuate, harm due to their focus on gender or sexuality. Inclusive models are advantageous because they add nuance to the different etiologies and forms of IPA that can be experienced by LGBTQIA+ people, such as denying someone’s gender/sexuality, which is compounded by additional constraints on help-seeking, such as limited access to formal services.

### Practical Implications for Minimizing Help-Seeking Barriers for LGBTQIA+ People

Our results are consistent with the statistics that LGBTQIA+ people experience high rates of IPA (Ministry of Justice, 2022), but illustrate that heightened rates of IPA are plausibly connected to the inaccessibility of education, prevention, and intervention for LGBTQIA+ people and do not support interpretations that this group is inherently more aggressive. The survey results (Study 1a) and interviews (Study 1b) stressed the additional harms that can be experienced in seeking support for IPA, including compounded stigma, feelings of shame and weakness, and fears of not being believed due to one’s gender/sexuality. Together, this suggests that the barriers faced by LGBTQIA+ people limit the prevention and intervention of IPA behaviors within the LGBTQIA+ community. A key aspect in overcoming barriers to help-seeking for IPA is telling people how to seek help and funding the sources that provide explicitly safe LGBTQIA+ support, rather than just telling people that they *should* seek support. Furthermore, increasing LGBTQIA+ people’s knowledge of the stages to expect within the help-seeking process can mitigate barriers surrounding uncertainty, such as letting people know how they can receive support without reporting their partner. Finally, the capacity for formal sources of support to effectively communicate knowledge about the help, as well as their accessibility to the LGBTQIA+ community, requires first building trust with the LGBTQIA+ community. One possibility, as highlighted in the interviews in Study 1b, is to have specialized services *for* LGBTQIA+ people that are run *by* LGBTQIA+ people, in addition to existing services that can increase their flexibility while still having high LGBTQIA+ competency (e.g., being responsive and gender-inclusive) (Dixon et al., 2023).

### Strengths, Limitations, and Future Directions

The context of the current research on societal heteronormativity was Aotearoa New Zealand—a country that is relatively accepting of the LGBTQIA+ community (Treharne & Adams, 2017). We illustrated that in this context, even when sources of support are available (e.g., Shine, Women’s Refuge), help-seeking was limited for the LGBTQIA+ community and people identified heteronormative structures that they believed would exclude them from help. However, in contexts where resources and support for IPA are less available (e.g., a lack of trained providers or funding) (Kanougiya et al., 2022) and/or where the acceptance of the LGBTQIA+ community is low (Rodríguez & Murtagh, 2022), LGBTQIA+ people will likely experience greater barriers to support outright. Accordingly, future research should examine how LGBTQIA+ people can still seek help for IPA when there are no specific pathways available and high discrimination toward the LGBTQIA+ community.

The relationship between societal heteronormativity and help-seeking for IPA will likely vary across cultures, given that cultural context informs gender roles and adherence to such roles (Cañete et al., 2022). For example, through colonization, the predominantly binary views of gender and sexuality in Western societies are held by many Indigenous peoples (Le Grice et al., 2022). As such, Indigenous people who hold different views of gender and sexuality will likely have distinct experiences of societal heteronormativity and thus requires participant-centric research methods to hold the expertise when covering unique and qualitatively distinct aspects of culture. In the current study, our aims considered how the bicultural context of Aotearoa New Zealand could uniquely shape societal attitudes and help-seeking, but we did not have the resources to compare different cultures or interview Takātapui people that could appropriately address that aim. We encourage future research to build consideration of how cultural contexts differ in heteronormativity, and how this, in turn, influences help-seeking for IPA within intersections of the LGBTQIA+ community.

## Conclusion

LGBTQIA+ people in Aotearoa New Zealand perceive barriers to help-seeking for IPA that were particularly present in formal supports that were seen as inaccessible and unsafe for LGBTQIA+ people. In addition, a small but significant link indicated that people who perceived greater heteronormativity felt more shame and embarrassment about seeking help for IPA. The help-seeking process is daunting for people who have experienced victimization, but belonging to a community that does not adhere to heteronormative ideas of IPA magnifies the potential threat. Accordingly, theory and practice must work to combat processes that create additional challenges for people seeking support for their experiences of victimization.

## Supplemental Material

sj-docx-1-jiv-10.1177_08862605241270045 – Supplemental material for A Mixed Methods Study of Barriers to Help-Seeking for Intimate Partner Aggression in the LGBTQIA+ Community Supplemental material, sj-docx-1-jiv-10.1177_08862605241270045 for A Mixed Methods Study of Barriers to Help-Seeking for Intimate Partner Aggression in the LGBTQIA+ Community by Chelsea R. D’Cruz, Matthew D. Hammond and Louise Dixon in Journal of Interpersonal Violence
