# Supplementary material for: A Mixed Methods Study of Barriers to Help-Seeking for Intimate Partner Aggression in the LGBTQIA+ Community
Source: J Interpers Violence. 2024 Aug 26;40(9-10):2163–87. doi: 10.1177/08862605241270045 (PMC11951454; doi:10.1177/08862605241270045)
Supplement: sj-docx-1-jiv-10.1177_08862605241270045 – Supplemental material for A Mixed Methods Study of Barriers to Help-Seeking for Intimate Partner Aggression in the LGBTQIA+ Community [file sj-docx-1-jiv-10.1177_08862605241270045.docx]

**A Mixed Methods Study of Barriers to Help-Seeking for Intimate Partner Aggression in the LGBTQIA+ Community – Supplementary Materials**

**Table of Contents**

[Table S1: Principle Component Analysis of Barriers to Support Scale 2](#_Toc167184003)

[Table S2: Descriptive Statistics and Correlations Across Primary Measures of Study 1a 4](#_Toc167184004)

[Table S3: Study 1b Interview Questions 5](#_Toc167184005)

# Table S1

Principle Component Analysis of Barriers to Support Scale

| Barriers to help-seeking item | Component | |  | |
| --- | --- | --- | --- | --- |
|  | 1 | 2 | Uniqueness |  |
| *Component 1: Other-focused barriers* |  |  |  |  |
| I would not know where to go for help | .501 |  | .748 |  |
| I would not believe that I would get proper help | .653 |  | .562 |  |
| I would not believe that this support source could help me | .638 |  | .586 |  |
| I would be afraid of the consequences of seeking help | .479 |  | .652 |  |
| I would be afraid that those I talk to would have negative attitudes towards me | .731 |  | .483 |  |
| I would be afraid to bring a bad name to my community if I disclosed what I was experiencing | .425 |  | .713 |  |
| I would not trust that my experiences would be kept confidential | .462 |  | .721 |  |
| I believe I would not be treated fairly because of my sexuality | .683 |  | .542 |  |
| I believe I would not be treated fairly because of my gender | .735 |  | .492 |  |
| *Component 2: Self-focused barriers* |  |  |  |  |
| I would be too embarrassed to discuss my problems with anyone |  | .568 | .596 |  |
| I would think my problem was one I should be able to cope with myself |  | .666 | .575 |  |
| I would not want any help |  | .479 | .790 |  |
| I would be ashamed to show others how troubled I was |  | .661 | .538 |  |
| Privacy is important to me, and I would not want other people to know about my problems |  | .502 | .717 |  |
| I’d feel better about myself knowing I didn’t need help from others |  | .694 | .542 |  |
| It would seem weak to ask for help |  | .698 | .528 |  |
| I would not want to appear weaker than my peers |  | .641 | .575 |  |

*Note.* Oblimin rotation was used.

# Table S2

Descriptive Statistics and Correlations Across Primary Measures of Study 1a

|  |  |  | *Formal Barriers* | | *Informal Barriers* | |
| --- | --- | --- | --- | --- | --- | --- |
|  | N | M *(SD)* | Self-focused | Other-focused | Self-focused | Other-focused |
| Perceived Heteronormativity | 359 | 3.39 (1.48) | .14* | .06 | .12* | -.01 |
| *Formal Barriers* |  |  |  |  |  |  |
| Self-focused | 318 | 4.38 (1.07) | - | .38** | .66** | .35** |
| Other-focused | 317 | 4.17 (0.99) |  | - | .33** | .57** |
| *Informal Barriers* |  |  |  |  |  |  |
| Self-focused | 323 | 4.37 (1.12) |  |  | - | .45** |
| Other-focused | 322 | 4.24 (1.15) |  |  |  | - |

*Note.* Measures were assessed on 1 to 7 scales with midpoints of 4. * *p* < .05. ** *p* < .01

# Table S3

*Study 1b Interview Questions*

| Interview Questions |
| --- |
| 1. Have you heard of the term ‘heteronormativity’? |
| - 1. (If participant answers “yes”) Could you describe, in your own words, what heteronormativity means to you? |
| 1. Can you describe to me, what heteronormativity in society looks like to you? |
| 1. Do you think these kind of attitudes are common in Aotearoa? |
| - 1. Why might that be? |
| 1. Can you describe any unique perspectives you think New Zealander’s might have on societal heteronormativity? |
| 1. When Rainbow people experience IPA, do you think they’d be more likely to seek help from formal services, like police, or informal services, like friends and family? |
| - 1. Why do you think that is? |
| - 1. Do you think there’s anything in particular about being Rainbow that would change this process compared to people who aren’t part of the Rainbow community? |
| 1. Do you think there’s anything that might prevent Rainbow people seeking help from formal services? |
| 1. Do you think there’s anything that might prevent Rainbow people seeking help from informal services? |
| 1. If we were able to situate ourselves in an ideal world, is there anything you can think of that might help Rainbow people report their experiences of IPA? |
| 1. Is there anything that might help Rainbow people access services that could help with these experiences? |
| 1. Do you think there’s anything specific that current services or agencies might be able to do in order to be more friendly to Rainbow people trying to report or get help for their experiences with IPA? |
| - 1. Would they need a different approach to how they respond to non-Rainbow people? |
| 1. Do you think there’s anything that we haven’t discussed that might facilitate Rainbow people seeking help? |
| - 1. That could be something that we see in society, or something the individual themselves might do differently? |
| 1. Is there anything else you’d like to add or discuss in relation to this? |

*Note:* other questions to follow-up or elaborate on a point were asked if necessary.
